# Supplementary material for: Computational and experimental exploration of statin and statin-like compounds as potential treatment of schistosomiasis
Source: PLoS Negl Trop Dis. 2025 Sep 12;19(9):e0013524. doi: 10.1371/journal.pntd.0013524 (PMC12448999; doi:10.1371/journal.pntd.0013524)
Supplement: S1 Table — (DOCX) [file pntd.0013524.s001.docx]

**S1 Table. Docking Scores and Binding Free Energy (MMGBSA) Estimates of Statins and Their Structural Analogues Grouped by Parent Compound**

|  | Structure/Name | Docking Score | MMGBSA |
| --- | --- | --- | --- |
|  | Lovastatin   | -7.11 | -31.12 |
|  |  | -7.92 | -31.10 |
|  |  | -7.61 | -39.96 |
|  |  | -7.34 | -20.87 |
|  |  | -7.32 | -17.47 |
|  |  | -7.23 | -50.91 |
|  |  | -7.21 | -23.99 |
|  |  | -7.12 | -22.14 |
|  |  | -7.05 | -42.47 |

| S/N | Structure /Name | Docking score | MMGBSA |
| --- | --- | --- | --- |
|  |   Pitavastatin | -8.448 | -34.112 |
|  |  | -7.97 | -31.09 |
|  |  | -7.90 | -30.63 |
|  |  | -7.88 | -50.32 |
|  |  | -7.87 | -34.74 |
|  |  | -7.85 | -24.52 |
|  |  | -7.82 | -28.17 |
|  |  | -7.74 | -23.80 |
|  |  | -7.7 | -35.38 |
|  |  | -7.52 | -28.02 |
|  |  | -7.34 | -27.17 |
|  |  | -7.15 | -27.10 |
|  |  | -7.03 | -24.73 |
|  |  | -7.01 | -15.47 |

| S/N | Structure/Name | Docking score | MMGBSA |
| --- | --- | --- | --- |
|  | Pravastatin   | -10.886 | -20.48 |
|  |  | -8.88 | -43.82 |
|  |  | -8.761 | -32.70 |
|  |  | -8.28 | -26.89 |
|  |  | -8.24 | -31.65 |
|  |  | -7.98 | -21.07 |
|  |  | -7.89 | -33.40 |
|  |  | -7.84 | -21.83 |
|  |  | -7.83 | -27.62 |
|  |  | -7.82 | -27.87 |
|  |  | -7.82 | -22.4 |
|  |  | -7.77 | -23.89 |
|  |  | -7.68 | -22.93 |
|  |  | -7.63 | -18.26 |
|  |  | -7.63 | -20.60 |
|  |  | -7.58 | -37.27 |
|  |  | -7.50 | -49.43 |

| S/N | Structure/Name | Docking score (kcal/mol) | MMGBSA  (kcal/mol) |
| --- | --- | --- | --- |
|  |   Fluvastatin | -9.44 | -49.37 |
|  |  | -9.28 | -20.668 |
|  |  | -9.12 | -18.79 |
|  |  | -9.01 | -29.40 |
|  |  | -9.02 | -29.74 |
|  |  | -8.73 | -13.41 |
|  |  | -8.72 | -21.99 |
|  |  | -8.7 | -24.72 |
|  |  | -8.49 | -14.85 |
|  |  | -8.29 | -23.10 |
|  |  | -8.22 | -36.32 |
|  |  | -8.21 | -20.30 |
|  |  | -7.933 | -27.11 |
|  |  | -8.18 | -18.44 |
|  |  | -8.18 | -47.15 |
|  |  | -8.14 | -30.62 |
|  |  | -8.11 | -13.18 |
|  |  | -8.01 | -28.01 |
|  |  | -8.03 | -24.54 |
|  |  | -7.82 | -27.54 |
|  |  | -7.79 | -21.74 |
|  |  | -7.78 | -25.35 |
|  |  | -7.76 | -26.33 |
|  |  | -7.68 | -23.04 |
|  |  | -7.62 | -30.66 |
|  |  | -7.61 | -35.41 |
|  |  | -7.59 | -21.62 |
|  |  | -7.57 | -25.42 |
|  |  | -7.51 | -37.74 |

This next section includes extended molecular interaction analyses for the statin analogues studied. Specifically, 2D interaction diagrams illustrating hydrogen bonding, hydrophobic contacts, and electrostatic interactions between each ligand and the *Sm*HMGR active site are provided for Pitavastatin, Fluvastatin, Pravastatin, and Lovastatin groups. These figures support the docking and binding free energy results presented in the main manuscript by offering visual insights into residue-level interactions. They are included here to complement the discussion while maintaining clarity in the main text.
