# Supplementary material for: Computational and experimental exploration of statin and statin-like compounds as potential treatment of schistosomiasis
Source: PLoS Negl Trop Dis. 2025 Sep 12;19(9):e0013524. doi: 10.1371/journal.pntd.0013524 (PMC12448999; doi:10.1371/journal.pntd.0013524)

***S1 Fig. 2D Interaction Diagrams of Pitavastatin and Its Derivatives with SmHMGR Active Site Residues***


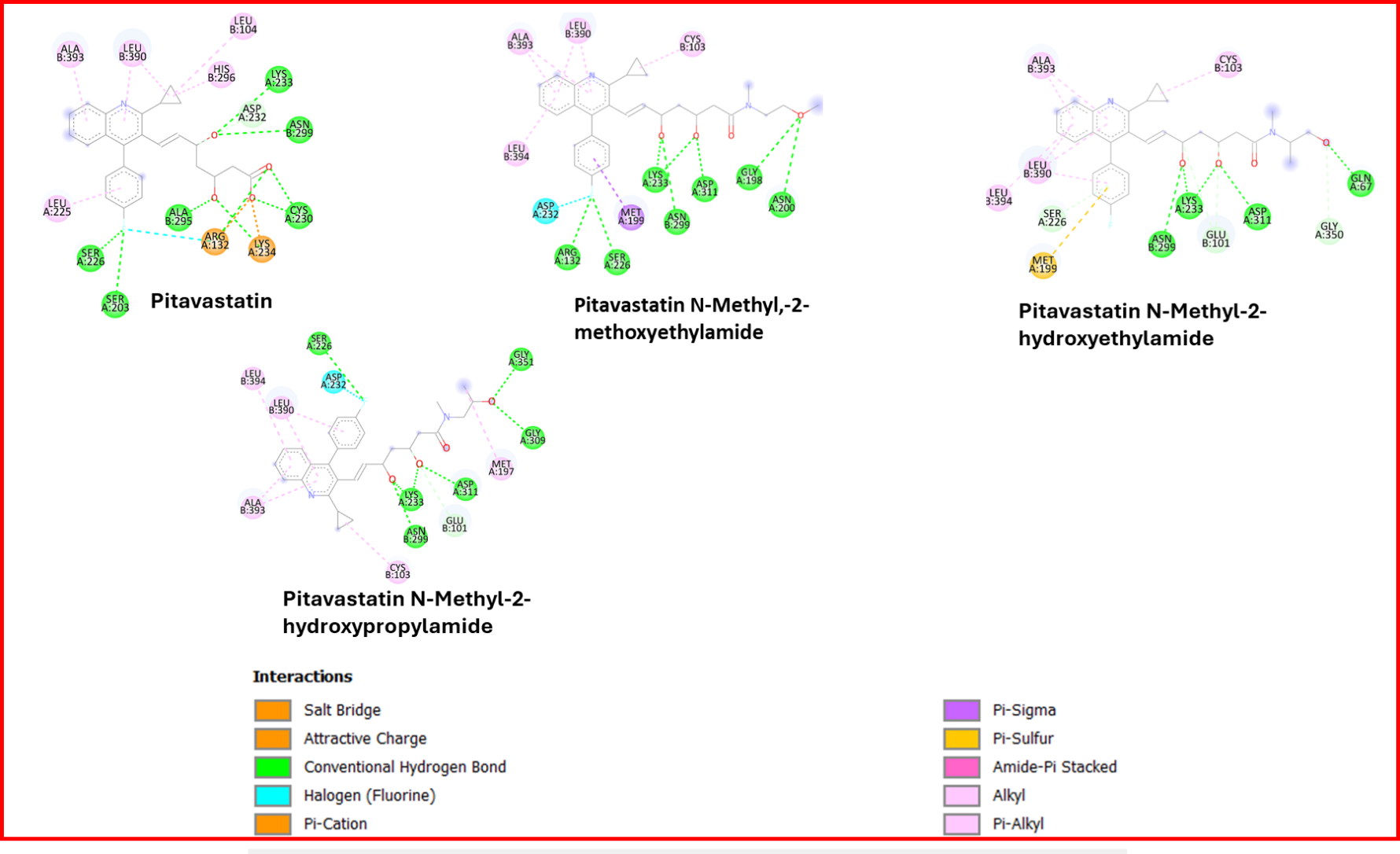

Supplement: S1 Fig — (DOCX) [file pntd.0013524.s002.docx]
