# Supplementary figures and images for: Computational and experimental exploration of statin and statin-like compounds as potential treatment of schistosomiasis
Source: PLoS Negl Trop Dis. 2025 Sep 12;19(9):e0013524. doi: 10.1371/journal.pntd.0013524 (PMC12448999; doi:10.1371/journal.pntd.0013524)

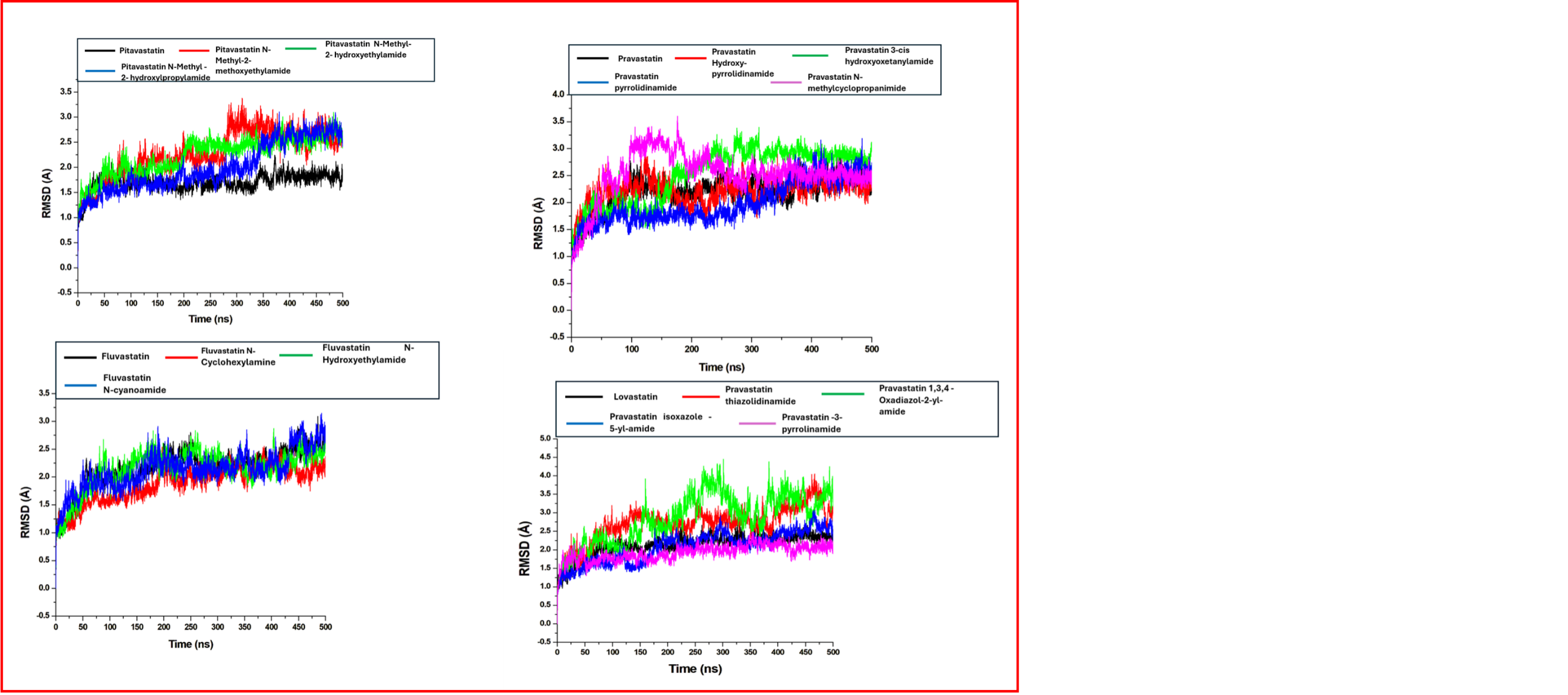


***S5 Fig. Structural Stability of SmHMGR Complexes with Statins and Their Top Derivatives***

Supplement: S5 Fig — (DOCX) [file pntd.0013524.s006.docx]
