# Supplementary material for: Computational and experimental exploration of statin and statin-like compounds as potential treatment of schistosomiasis
Source: PLoS Negl Trop Dis. 2025 Sep 12;19(9):e0013524. doi: 10.1371/journal.pntd.0013524 (PMC12448999; doi:10.1371/journal.pntd.0013524)
Supplement: Attachment — Submitted filename: Reviewer comments_PNTD.pdf [file pntd.0013524.s007.pdf]

Reviewer comments on the study titled: Computational and experimental exploration of statin and statin-like compounds as potential treatment of schistosomiasis.

Overview

Authors are reporting a classical approach in the drug discovery of schistosomicidal compounds, focusing their efforts to find active substances that could disturb the mevalonate pathway of S. mansoni by targeting SmHMGR. Furthermore, authors are focusing on statins and statin-like (modified statins) as potential candidates to become therapeutic options to praziquantel. The study adds important value to the field, reporting results for not yet tested compounds while explores the already paved avenue that shows statin-like compounds as promising alternatives to tackle schistosomiasis.

Introduction

Clear, well-structured and with sufficient resources to show the reader the problem their study is challenging.

Methodology

Classical combination of in silico approaches to prioritise a subset of compounds to be tested against different life stages of S. mansoni.

Line 104 – please, correct the way references 28 and 29 are being displayed.

Question: why did the authors prefer to use MODELLER instead of AlphaFold? (this is out of curiosity, not for promoting one methodology against the other!!)

Subsection 2.2 - Question: quality of binding was assessed by only filtering those with lower scores? Were there any visual inspection and analysis of individual interactions to ensure the inverse docking was a validated starting point to assess the poses of other molecules of interest?

Subsection 2.2 – Question: could the authors please report all the parameters used to conduct the molecular docking using AutoDock Vina?

Subsection 2.2 – Question – could the authors please clarify why did the authors use only AD Vina to perform the inverse docking and then compare AD Vina to Glide during VS process?

Line 264 – “the production MD” – is it a typo? If so, please correct.

Line 303 – this is the first instance where the acronym NTS appears, however it is defined only in line 308. Please, fix it accordingly.

Results

Fig 4A – did the authors use Glide to visualise the predicted ligand-binding site interactions? If so, please cite it accordingly.

Section 3.4 – could the authors please expand a bit more on the usage of fragment- and lead-likeness filters? I'm asking this because at this point it's still not clear if optimising fragments was part of the main goal of the study. (Please, do ignore this comment if explained later in the paper, as I'm commenting while reading through the document.) – As shown in Table 3, no fragment- or lead-like analogues were retrieved, so my question remains: why using (and reporting) such filtering criteria? Would using only Ro5 filtering lead to the same result?

Further, could the authors please report in their response how many of those 10^{12} chemicals were filtered out just by using the similarity cutoff of 0.7 with parent statins as reference molecules? Were those 11,959 initial pool analogues (Line 475) the result of this filtering?

Lines 508-9 – could the authors please clarify a bit more how and why an AUC of 72.663 was obtained? I was under the impression that this value was a ROC AUC (bounded between 0 and 1).

Figure 8 – is this color-code the regular, default one? Or did the authors color the residues in the same way showed previously in the manuscript?

Line 587 – please, could the authors ensure the use of acronyms consistently?

Conclusion

Lines 784-785 – authors state that computational approaches "revealed interactions that guided the identification and optimisation of statin analogues" – which is misleading. Authors did not do any type of optimisation of structure features neither synthesised new statin-like derivatives. Could authors please clarify this statement?

Lines 788-789 – could the authors please expand a bit more on which "substantial morphological and behavioural changes in adult worms" were observed?

Comment: it would add value to this work including a deeper discussion on the impacts of the high concentration tested for each statin-like against NTS and adult worms, bearing in mind both further steps of drug development pipeline and potential interaction of these analogues with human HMGR, given its similarity with SmHMGR.
