# Supplementary material for: Computational and experimental exploration of statin and statin-like compounds as potential treatment of schistosomiasis
Source: PLoS Negl Trop Dis. 2025 Sep 12;19(9):e0013524. doi: 10.1371/journal.pntd.0013524 (PMC12448999; doi:10.1371/journal.pntd.0013524)
Supplement: Attachment — Submitted filename: Response to Reviewers.docx [file pntd.0013524.s008.docx]

**Computational and Experimental Exploration of Statin and Statin-like Compounds as Potential Treatment of Schistosomiasis.**

We thank the editor and reviewers for their thorough and constructive evaluation of our manuscript. We have carefully addressed all comments and revised the manuscript accordingly to improve its clarity, rigor, and scientific framing. Below, we provide a detailed point-by-point response to each comment. We trust that our revisions have resolved the major concerns raised; however, we remain fully open to any further feedback or additional revisions that the editor or reviewers may recommend.

**Response to Reviewers**

| **Editor’s comment** | **Rebuttal** |
| --- | --- |
| Please upload all main figures as separate Figure files in .tif or .eps format. | We have uploaded all main figures in tif format |
| We have noticed that you have uploaded Supporting Information files, but you have not included a list of legends. Please add a full list of legends for your Supporting Information files after the references list. | The supporting file figure legends have been added at the end of the reference list |
| Please amend your detailed Financial Disclosure statement. This is published with the article. It must therefore be completed in full sentences and contain the exact wording you wish to be published  State what role the funders took in the study. If the funders had no role in your study, please state: "The funders had no role in study design, data collection and analysis, decision to publish, or preparation of the manuscript.  If any authors received a salary from any of your funders, please state which authors and which funders. If you did not receive any funding for this study, please simply state: u201cThe authors received no specific funding for this work.u201d | This study was funded by the United Kingdom Royal Society (Grant number: NIF/R1/211737) awarded to KFP-O. The funders had no role in study design, data collection and analysis, decision to publish, or preparation of the manuscript.  KFP-O received a salary during this study period through the Royal Society grant. No other authors received salaries from the funder. |
| We have amended your Competing Interest statement to comply with journal style. We kindly ask that you double check the statement and let us know if anything is incorrect. |  |

| **Reviewer 1 Comment** | **Response** |
| --- | --- |
| The objective of the study was to use a computational pipeline to systematically identify and optimize SmHMGR inhibitors. The computational part of the study, although extensive, lacks objectivity and it is reflected on the modest antischistosomal activities found for the final compounds selected by the computational pipeline. The computational methodology is based on structure-based drug design tools but the experimental validation is performed by phenotypic assays. This causes an important gap between the computational predictions and computational results. Authors didn't discuss this important aspect of their study. Are such modest experimental findings the result of poor performance of the computational methodology or the inherent hurdles in translating target-based predictions to in vitro biological activities on whole organisms? | We sincerely thank the reviewer for this insightful and constructive comment. We agree that there is a recognised gap between structure-based predictions and phenotypic outcomes, and we have now discussed this in more detail in lines 809 -811 and 826-835  This study was designed as an exploration of statin-like compounds as potential inhibitors of *Sm*HMGR. As stated in the title, introduction (Lines 91 -102) and throughout the manuscript, the primary goal was to use a computational framework to systematically identify potential *Sm*HMGR inhibitors and assess their experimental relevance. We did not expect a predefined result, nor did we anticipate a linear correlation between docking scores and whole-organism efficacy.  Indeed, the modest activity observed in the phenotypic assays could be due to several factors outside the scope of structure-based design, including issues related to compound permeability, metabolic stability, target engagement in a complex parasite system, or limited bioavailability *in vitro*. These are common and well-documented hurdles in translating target-based predictions into phenotypic effects in schistosomiasis and other helminth models.  Moreover, early-stage hits from exploratory docking campaigns often require multiple cycles of hit-to-lead optimisation before any significant potency emerges in phenotypic assays. As such, we believe the modest activity reflects the real-world complexity of whole-parasite systems rather than a failure of the computational approach itself.  We have now included this clarification to better frame the scope and limitations of the work. We hope this addresses the reviewer’s concern and provides a more balanced understanding of the predictive and exploratory roles of computational modelling in neglected disease drug discovery. |
| Authors should define “activity” early on the manuscript. It seems form pieces of information scattered across the manuscript that “activity” is the percentage of dead parasites. In this case, authors should clearly define how parasite death was assessed. | In line 345 -346 we describe how activity is defined. It is not only dead parasites but a viability scale is used to evaluate drug effects on worms. *Parameters such as motility, viability, and morphological changes were scored using a pre-defined scale. NTS viability was assessed after 72 hours using similar criteria.*  In the revised manuscript a reference was provided so that the reader can obtain further information |
| Topic 2.1: What changes from each of the 140 models to the other? | We thank the reviewer for raising this insightful question. The set of 140 *Sm*HMGR homology models was generated to comprehensively sample conformational variability, particularly around the binding site, where loop flexibility can influence ligand accommodation.  While the overall fold and core structure were conserved across models, variations were observed primarily in the backbone and side-chain orientations within flexible loop regions near the active site. These subtle differences represent alternative low-energy conformations rather than large structural deviations, and were critical for capturing receptor flexibility in our structure-based screening.  To ensure reliability, approximately 40 models were excluded due to modelling artefacts such as disrupted secondary structures, poor geometry in functionally relevant loops, or steric clashes that would compromise docking accuracy. The remaining 100 models provided a robust ensemble for downstream virtual screening and binding free energy estimations. |
| Topic 2.2: Is atorvastatin a known ligand of SmHMGR. What makes it a good template to search for other inhibitors of the enzyme? | We thank the reviewer for this question. Atorvastatin is not a known ligand of *Sm*HMGR but is a validated inhibitor of human HMGR. In our workflow, atorvastatin was used solely in the inverse docking step to prioritise relevant receptor conformations, as it was co-crystallised with the human HMGR template (PDB ID: 1HWK) used for homology modelling. This enabled us to assess how well each model could accommodate a known HMGR ligand.  It is important to clarify that atorvastatin was not used to search for analogues of other statins. Each parent statin, including atorvastatin, pitavastatin, rosuvastatin, and others, was individually submitted to the Infinisee database to identify structurally similar analogues. This ensured that each analogue set was specific to its parent compound. These distinctions are already detailed in Sections 2.1 to 2.2 and 3.4 of the manuscript. |
| Line 128: “atorvastatin and each SmHMGR model were optimally formatted in .pdb files”. What exactly this means? What were the preparation steps? | We thank the reviewer for pointing this out. The sentence has now been revised for clarity. Lines 139 -140. What was meant is that both the protein models and the ligand were simply saved in .pdb format as required by the docking software. No specific structural preparation steps were performed at this stage beyond format conversion. |
| Lines 197-198: Please clarify. What do the authors mean by "stereochemical variability"? How 10 stereoisomers can be generated per ligand? | We thank the reviewer for this observation. By “stereochemical variability,” we refer to the generation of possible stereoisomers for ligands with undefined chiral centres using LigPrep. Lines 210 -212. This tool was configured to generate up to 10 stereoisomers per ligand based on chiral centres that were not explicitly defined in the original structure files. We have now clarified this in the manuscript. |
| Lines 203-204: Specify what algorithm was used with what restraints, number of minimization steps and convergence criteria. | We thank the reviewer for this helpful suggestion. Energy minimisation of the receptor was performed using the Protein Preparation Wizard in the Schrödinger Suite, employing the OPLS3e force field. A restraint of 0.30 Å RMSD was applied to heavy atoms to preserve the original geometry. The minimisation proceeded until the energy gradient converged below 0.01 kcal/mol/Å or after a maximum of 3000 steps. We have now added these details to the revised manuscript in lines 216 -219. |
| Topic 3.2 (Inverse docking): Are there any published methodological papers supporting this claim? I'm not convinced of the utility of this approach. The major flaw for me is that you are using a docking protocol, which has not been validated to select target models. | We thank the reviewer for raising this important point. We appreciate the opportunity to clarify that inverse docking is a recognised and widely applied methodology in structure-based drug discovery, with multiple published studies supporting its use for identifying potential protein targets and prioritising receptor conformations for ligand binding.  In our case, inverse docking was not used for target fishing or off-target identification. Rather, it was employed to evaluate how a known ligand - atorvastatin interacts with an ensemble of *Sm*HMGR models representing binding site flexibility. This strategy aligns with established practices in ensemble docking, where ligand compatibility with different receptor conformers is assessed to select the most relevant structures for downstream virtual screening.  Several studies have demonstrated the utility and reliability of inverse docking in similar contexts:  Chen and Zhi (2001) introduced the concept of ligand-protein inverse docking to identify potential targets for small molecules.  Platforms such as TarFisDock (Li et al., 2006), TargetHunter (Wang et al., 2013), and idTarget (Wang et al., 2012) have built upon this concept and been applied to drug repurposing, toxicity prediction, and target validation.  Pinzi and Rastelli (2019) provided a comprehensive review of docking methods, including inverse docking, highlighting its increasing relevance and validation in drug discovery pipelines.  In our workflow, this approach served to narrow down the *Sm*HMGR conformers to those most compatible with ligand binding before applying more resource-intensive screening. We have revised Section 2.2 of the manuscript to reflect this rationale and have cited the supporting literature accordingly. Lines 133 -`138 |
| Line 353 and 390: Define "strong". Please use RMSD metric. Define "robust" and "stable", authors should try to be as objective as possible using quantitative strucutral parameters to support their observations instead of vague adjectives. | The vague adjectives have been removed in line 361 and the statements in line 396 have been modified |
| Fig 1: The last G (human) and D (SCHMA) are highlighted but are distinct. There is also a V-L pair between 240 and 300 | This has been corrected |
| Line 403: What is rec061? | We thank the reviewer for pointing this out. “rec061” was an internal labelling system used before relabelling the models as recA, recB, and recC. This has now been corrected in the manuscript. Line 408 |
| The conclusions are, for the most part, not supported by the data. For instance, authors state in line 774 that "analogues of pitavastatin (Table 9) exhibited promising activity". Based on what premises? What is the target activity/potency profile for an initial hit in schistosomiasis drug discovery? | In line 341-342 we define a significant as 66% activity. A threshold of 60-70% is commonly used to define hits for a tested concentration |
| Authors also claim to have "optimized" statin analogues but optimization is the process where one start from an initial hit and then optimize the chemical structure to improve biological activities. But activity of the parent compound was not known. | We sincerely thank the reviewer for this correction. We acknowledge that the term “optimised” was not appropriately used in this context, as we did not perform any structural modifications or lead optimisation steps. Our intention was to prioritise compounds using a computational pipeline, not to optimise them chemically. We have now removed all references to “optimisation” from the manuscript to accurately reflect the scope of the work. |
| Overall, authors use a large part of the manuscript to describe computational results that ended up not performing as well as expected in terms of experimental validation. The manuscript would gain a lot in readability if the authors made an effort to only described the most relevant results and the ones directly relating to the experimental results. Everything else should be move to supplementary material.  In Summary, this study aimed to employ structure-based drug design computational tools to identify new inhibitors of SmHMGR, a rising target for antischistosomal drug discovery. The study is greatly limited by the fact that experimental validation of the results are obtained straight from phenotipc assays on in vitro cultivated parasites without first testing on the molecular target. Althogh this approach is feasible, authors seem to completely ignore it in their discussion and make overstatements about the performance of the computational approach. In my view there is interesting data woth publishing in this manuscript but should be majorly  reorganized to become more concise and focused on the discussion of the experimental results in view of the limitations of the computational approach. | We thank the reviewer for these thoughtful comments. We would like to respectfully clarify that this study was designed as an exploration of statin and statin-like compounds as potential inhibitors of *Sm*HMGR, as stated in the title and throughout the manuscript. The computational framework was employed not to predict a guaranteed outcome, but to systematically investigate plausible interactions and generate hypotheses that could inform future optimisation and experimental refinement.  While we fully understand the reviewer’s suggestion to limit the manuscript to results that correlate directly with the phenotypic assays, our intent was to present the complete exploration path, including findings that did not translate directly into phenotypic success, so that other researchers can better understand the challenges and opportunities of targeting *Sm*HMGR in schistosomiasis drug discovery.  We acknowledge the reviewer’s point on the absence of direct molecular target validation. Given resource limitations and technical constraints, our experimental validation proceeded via phenotypic screening of schistosomula and adult worms. We have now clearly acknowledged this limitation and the potential consequences in Lines 827 to 836, ensuring that the study’s scope and limitations are accurately communicated.  To avoid overstatement, we have carefully revised language throughout the manuscript, especially in the discussion and conclusion sections, to reflect the exploratory nature of the study and avoid any implication that the computational method was conclusive or fully validated by the experimental results.  We remain grateful for this perspective and believe that with these clarifications, the manuscript now more clearly communicates its intent, methodology and limitations. |
| Line 65: authors should more accurately discuss this PZQ limitations since the latter is actually able to kill schistosomula in vitro at least and consider the juvenile stage as well. | We thank the reviewer for this important clarification. We agree that praziquantel (PZQ) has demonstrated activity against schistosomula under certain in vitro conditions, although its efficacy against juvenile stages remains suboptimal and variable depending on timing and assay design. We have now revised the manuscript to reflect this more nuanced view of PZQ’s limitations.  While praziquantel (PZQ) remains the mainstay of schistosomiasis treatment, its efficacy against juvenile parasites is limited and variable. Although in vitro studies have shown that PZQ can kill schistosomula under specific conditions, it is generally less effective against early developmental stages in vivo, contributing to concerns about reinfection and treatment gaps in endemic populations . This has been revised in lines 64-68 |
| Lines 80-81: Discuss similarity with human homologue and discuss potential selectivity issues. | We thank the reviewer for this important comment. Sequence alignment between *Sm*HMGR and the human HMGR template revealed a 52.33% similarity in their catalytic domains, with key binding site residues conserved (as shown in Figure 1). We acknowledge that this similarity may pose potential challenges for selectivity. Accordingly, we have now addressed this issue in the Conclusion by noting that future studies should include selectivity assessments to ensure minimal off-target effects on the human homologue. Lines 823 -824 |

**Reviewer 2**

| **Reviewer 2 comment** | **Rebuttal** |
| --- | --- |
| Line 104 – please, correct the way references 28 and 29 are being displayed. | This has been corrected in lines 112 |
| Question: why did the authors prefer to use MODELLER instead of AlphaFold? (this is  out of curiosity, not for promoting one methodology against the other!!) | We thank the reviewer for this thoughtful question. We selected MODELLER because it enabled us to generate a large ensemble of *Sm*HMGR models with controlled conformational variability, particularly around the binding site. This was important for our workflow, which incorporated receptor flexibility across multiple models while maintaining rigid-receptor docking within each individual run.  At the time of our study, AlphaFold was optimised for producing a single, high-confidence structural prediction. Its standard implementation does not currently support the generation of multiple conformers or targeted sampling of binding site flexibility, which are critical for ensemble docking approaches. For this reason, MODELLER was better suited to our objective.  We acknowledge the strengths of AlphaFold and are open to exploring its integration into future studies, where appropriate. |
| Subsection 2.2 - Question: quality of binding was assessed by only filtering those with lower scores? Were there any visual inspection and analysis of individual interactions to ensure the inverse docking was a validated starting point to assess the poses of other molecules of interest? | We thank the reviewer for raising this important point. In addition to score-based filtering, we performed visual inspection of the top-ranked poses to assess binding mode plausibility, including analysis of hydrogen bonding, hydrophobic contacts, and correct pocket orientation. We have now clarified this in the subsection 2.2 = Lines 151 -155 |
| Subsection 2.2 – Question: could the authors please report all the parameters used to conduct the molecular docking using AutoDock Vina? | We thank the reviewer for this important point. We have now added the full AutoDock Vina parameters to the manuscript in lines 140 -156 |
| Subsection 2.2 – Question – could the authors please clarify why did the authors use only AD Vina to perform the inverse docking and then compare AD Vina to Glide during VS process? | We thank the reviewer for raising this important point. AutoDock Vina was selected for the inverse docking step because it provides greater flexibility for screening a single ligand across multiple protein models using automated workflows. The open-source nature of Vina made it feasible to develop a custom script to efficiently perform this process, allowing us to compare the binding of atorvastatin across our ensemble of *Sm*HMGR models.  Glide, in contrast, is primarily optimised for conventional virtual screening workflows, where large ligand libraries are docked against a single receptor grid. To the best of our knowledge, Glide does not natively support inverse docking or large-scale scripting of ligand-to-receptor loops within its standard framework. Performing inverse docking with Glide would typically require the manual generation of multiple receptor grids and separate docking runs for each, which can be technically challenging and computationally cumbersome when dealing with large numbers of protein models.  For the virtual screening phase of our study, we selected Glide because of its recognised precision in ligand ranking and pose prediction, particularly for focused compound libraries against a defined target. This two-step approach, using AutoDock Vina for the model selection phase followed by Glide for high-accuracy ligand screening, enabled us to balance computational efficiency with predictive rigour in the later stages of the workflow. |
| Line 264 – “the production MD” – is it a typo? If so, please correct. | This has been reworded as the production phase of the MD simulation - line 274 |
| Line 303 – this is the first instance where the acronym NTS appears, however it isdefined only in line 308. Please, fix it accordingly. | This has been fixed in lines 310-311 |
| Fig 4A – did the authors use Glide to visualise the predicted ligand-binding site interactions? If so, please cite it accordingly. | It was visualized using Discovery Studio and this has been referenced in lines 152 |
| Section 3.4 – could the authors please expand a bit more on the usage of fragment- and lead-likeness filters? I’m asking this because at this point it’s still not clear if optimising fragments was part of the main goal of the study. (Please, do ignore this comment if explained later in the paper, as I’m commenting while reading through the document.) –  As shown in Table 3, no fragment- or lead-like analogues were retrieved, so my question remains: why using (and reporting) such filtering criteria? Would using only Ro5 filtering lead to the same result? | We thank the reviewer for these thoughtful questions. Optimising fragments or lead-like compounds was not part of the main goal of this study, and we acknowledge that this has now been clarified following comments from both reviewers. Our primary objective was to identify and prioritise drug-like compounds, not to optimise chemical scaffolds or fragment hits.  We included fragment- and lead-likeness filters to assess the chemical space composition of the screened library, rather than to guide compound selection. This provided insight into the distribution of compound types within the library, as summarised in Table 3, which includes a column explicitly reporting the classification of each compound as fragment-like, lead-like, or drug-like. The purpose of this step was to understand the diversity of the input library and ensure transparency about its composition.  As shown in Table 3, no fragment- or lead-like analogues were present among the prioritised candidates. Even if such compounds had emerged during the filtering, our focus would have remained solely on drug-like candidates for downstream evaluation.  Regarding the second point, applying only Lipinski’s Rule of Five would likely have led to a similar outcome in this case, given the composition of the compound library. However, including fragment- and lead-like filters allowed us to explicitly demonstrate this and document the screening process comprehensively.  We have condensed this point and added it to line 452-457 |
| Further, could the authors please report in their response how many of those 10^12^chemicals were filtered out just by using the similarity cutoff of 0.7 with parent statins as reference molecules? Were those 11,959 initial pool analogues (Line 475) the result of this filtering? | We thank the reviewer for this question and are pleased to clarify. The 11,959 analogues reported in Line 486 - 487 represent the full output retrieved from the Infinisee database after applying the ECFP4 fingerprint search with a similarity cutoff of 0.7 against the parent statins |
| Lines 508-9 – could the authors please clarify a bit more how and why an AUC of 72.663 was obtained? I was under the impression that this value was a ROC AUC (bounded between 0 and 1). | We thank the reviewer for raising this important point. The AUC values reported in Section 3.5 represent percentage-based enrichment AUCs, consistent with standard practices in virtual screening and early enrichment analysis. For clarity, we have now rephrased the manuscript to express the AUC values as percentages alongside their decimal equivalents. For example, an AUC of 72.66% corresponds to 0.73 on the conventional 0 to 1 ROC AUC scale. This clarification has been applied consistently across AutoDock Vina, Glide SP, and Glide XP. Corrections a are found in Lines 519 -520, line 528 and lines 543 -545 |
| Figure 8 – is this color-code the regular, default one? Or did the authors color the residues in the same way showed previously in the manuscript? | We confirm that Figure 8 uses Chimera’s standard default colour scheme. |
| Line 587 – please, could the authors ensure the use of acronyms consistently? | We thank the reviewer for pointing this out. We have reviewed the manuscript to ensure that **Binding Free Energy (BFE)** is introduced in full upon first mention, and the acronym is used consistently thereafter. The term was expanded in **line 604 before the subsequent acronym use** |
| Lines 784-785 – authors state that computational approaches “revealed interactions that guided the identification and optimisation of statin analogues” – which is misleading. Authors did not do any type of optimisation of structure features neither synthesised new statin-like derivatives. Could authors please clarify this statement? | We have deleted the word optimization. We appreciate the reviewer for spotting this |
| Lines 788-789 – could the authors please expand a bit more on which “substantial morphological and behavioural changes in adult worms” were observed? | We agree with the reviewer and have deleted the word substantial as the activity against adult worms was around 50%. A decreased motility was observed which was included in the manuscript in lines 805-806 |
